# Using artificial intelligence to improve COVID-19 rapid diagnostic test result interpretation

**DOI:** 10.1073/pnas.2019893118

**Published:** 2021-03-05

**Authors:** David-A. Mendels, Laurent Dortet, Cécile Emeraud, Saoussen Oueslati, Delphine Girlich, Jean-Baptiste Ronat, Sandrine Bernabeu, Silvestre Bahi, Gary J. H. Atkinson, Thierry Naas

**Affiliations:** ^a^xRapid-Group, 13100 Aix en Provence, France;; ^b^Bacteriology-Hygiene Unit, Assistance Publique/Hôpitaux de Paris, Bicêtre Hospital, 94275 Le Kremlin-Bicêtre, France;; ^c^INSERM Public Health Research, UMR 1184, RESIST Unit Paris-Saclay University, Faculty of Medicine, 94270 Le Kremlin-Bicêtre, France;; ^d^Associated French National Reference Center for Antibiotic Resistance: Carbapenemase-Producing Enterobacteriaceae, 94270 Le Kremlin-Bicêtre, France;; ^e^Mini-Lab Project, Medecins Sans Frontière, 75019 Paris, France

**Keywords:** SARS-CoV-2, machine learning, smartphone application

## Abstract

Serological rapid diagnostic tests (RDTs) are widely used across pathologies, often providing users a simple, binary result (positive or negative) in as little as 5 to 20 min. Since the beginning of the COVID-19 pandemic, new RDTs for identifying SARS-CoV-2 have rapidly proliferated. However, these seemingly easy-to-read tests can be highly subjective, and interpretations of the visible “bands” of color that appear (or not) in a test window may vary between users, test models, and brands. We developed and evaluated the accuracy/performance of a smartphone application (xRCovid) that uses machine learning to classify SARS-CoV-2 serological RDT results and reduce reading ambiguities. Across 11 COVID-19 RDT models, the app yielded 99.3% precision compared to reading by eye. Using the app replaces the uncertainty from visual RDT interpretation with a smaller uncertainty of the image classifier, thereby increasing confidence of clinicians and laboratory staff when using RDTs, and creating opportunities for patient self-testing.

The ability to rapidly test for infectious disease is vital in most outbreaks. Rapid diagnostic tests (RDTs) or lateral flow immunoassays (LFIA) (1) are widely used to test for conditions as varied as pregnancy, malaria, legionella (antigen detection in urine), antibiotic resistance, SARS, H5N1 flu, and more recently, SARS-CoV-2 (2–4). These RDTs consist of a strip, coated with a specific antigen, upon which human fluid (blood, plasma, urine, or mucus) and a reagent buffer are placed. A “band” of color (indicating positive, negative, or invalid results) appears in 5 to 20 min (5). RDTs are simple to use and relatively cheap. Some are used as self-tests that do not require the assistance of medical personnel to draw venous blood. During the COVID-19 pandemic, RDTs have been used outside of health facilities in ambulatory or even “drive-through” testing centers. They allow large populations to be tested with minimal training and gather critical data to guide authorities as they navigate pandemic shutdown and reopening procedures [see, for example, the World Health Organization’s recommendations for malaria RDTs (6)].

As of June 2020, more than 176 SARS-CoV-2 serological RDTs had been developed (7–9). However, despite their simplicity, interpreting RDT test results is not always straightforward. Low antibody levels can produce results (bands) that are not clearly distinguished, tempting users to falsely read a test as negative and making interpretation highly subjective.

To improve SARS-CoV-2 RDT interpretation and diagnosis, we developed a smartphone application (xRCovid app) to interpret RDT results using an artificial neural network (ANN) (10). The ANN analyzes SARS-CoV-2 RDT test results by standardizing readings, identifying conformity between results, and enabling traceability to ultimately provide a clearer diagnosis (11, 12). The app’s diagnostic yield and overall performance were developed and evaluated across 11 available SARS-Cov-2 serological RDTs.

## Results and Discussion

Although more than a hundred RDTs are available on the market after 6 mo of the COVID-19 pandemic, recent publications have shown that their analytical performance varies greatly (8). All can identify the presence SARS-CoV2 antibodies, but the intensity of the band (and thus its ease of interpretation) is entirely dependent on the level of antibody present in the sample. The xRCovid smartphone application addressed this challenge by combining the high-resolution imaging capabilities of a smartphone camera with ANN image treatment to determine, read, and interpret RDT results. The xRCovid app’s simple interface displays a clear positive/negative outcome and provides information depending on the result (i.e., care-seeking guidance for positive results, precautionary measures for negative). Future iterations may eventually provide results directly to a managing physician. The whole analysis is performed on the device, and full privacy for the diagnostic result is guaranteed.

Two hundred and fifty sera from 159 PCR-confirmed SARS-CoV-2 patients (collected from 0 to 32 d after onset of symptoms) were tested with rapid serological tests and human reading (8). Control sera (*n* = 254) were retrieved from pre-COVID periods from patients. All samples were tested using rapid LFIA from 10 manufacturers (8).

**Fig. 1.**
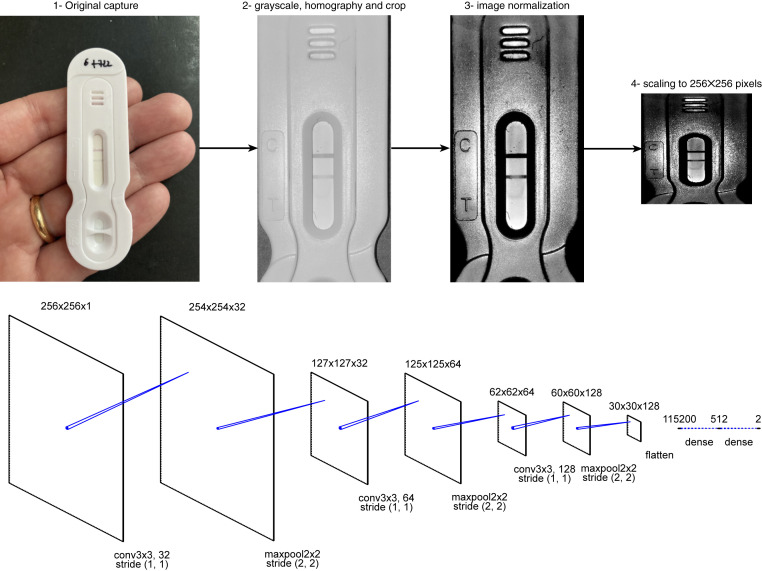
Image treatment (*Top*) and convoluted neural network (CNN) (*Bottom*) used. The image treatment extracts the grayscale image, crops it to the region of interest, normalizes the image, and squeezes it to a smaller dimension for the CNN. The CNN is a simple binary classifier that includes three convolution layers with max pooling and dropout regularization at 0.25, a dense layer with dropout regularization at 0.5 before the final dense layer.

The automated reading technology begins with a focused image of the RDT. A convoluted neural network (CNN), or simple image classifier, is used to identify the specific RDT being used. Image treatment is then applied: A planar homography is used to extract a straightened, cropped image of the RDT, and a second neural network identifies positivity/negativity (Fig. 1). Using machine learning, the same network architecture (10) was used to “train” the app with three different datasets. The first dataset identified the RDTs by brand name and model (*n* = 100 images of each RDT), although this step was omitted when a user opted to select the RDT from a drop-down menu. The second dataset trained the network to identify positivity or negativity, irrespective of the test brand or model (*n* = 1,000, 500 images each of positive and negative samples). A third dataset trained the ANN to read tests with two windows (and their corresponding positive or negative results), again regardless of RDT (*n* = 1,000, 500 images each of positive and negative samples). All models were trained to 99.5% sensitivity and 99.9% specificity. Data augmentation was used to improve the convergence of the machine learning models: a variation on the luminance (within ±10%) and the zoom factor (within ±40%). The image being converted to grayscale and normalized, the ANN is independent of color variations. This architecture was chosen for its greater tolerance to defects than a simpler support vector machine or straight image treatment. Importantly, all images were captured using fixed illumination by activating the smartphone flash during image capture. Using a constant light source avoided reflection and shadow for RDT windows with slanted edges, and enabled consistent readings between RDTs. Models were further used to read a large number of RDTs (*n* = 3,344) that they had not been trained for (Table 1). The relatively small number of false-positive (FP) and false-negative (FN) results allowed researchers to analyze the source of errors on a case-by-case basis.

**Table 1.** Prediction results from the neural network on 3,344 RDTs from 10 brands with respect to human reading

Model	TP	TN	FP	FN	Sensitivity, %	Specificity, %
Alco	5	19	0	0	100.0	100.0
Avioq	207	104	1	5	97.6	99.0
Biolidics	143	190	0	3	97.9	100.0
Biotime	18	7	0	0	100.0	100.0
Biosynex	63	126	0	1	98.4	100.0
NGBiotech Cassette	628	621	1	4	99.4	99.8
NGBiotech All in one	310	538	3	1	99.7	99.4
Nova	199	67	0	4	98.0	100.0
Realy	16	11	0	0	100.0	100.0
Solo Lab	16	9	0	0	100.0	100.0
Vedalab	18	6	0	0	100.0	100.0
Global	1,623	1,698	5	18	98.9	99.7

The true positives (TPs), true negatives (TNs), false positives (FPs), false negatives (FNs), sensitivity, and specificity are reported per device and for the whole set.

Ultimately, the automated reading technology’s high sensitivity and specificity with respect to the human reading baseline justified the use of simple classifier architecture. Out of 3,344 tested samples, only 18 FNs were observed and were due to low band contrast (15 out of 18 FNs for IgM and 3 out of 18 FNs for IgG). FP results (5 of 3,344 tested devices) occurred when markings (e.g., blood spots) were mistakenly interpreted by the ANN. Complete sample hemolysis in a test with a poorly functioning barrier also prevented researchers from reading tests (when an entire window was stained by hemoglobin, partially or completely covering the band detection area).

The model yields class output probabilities between 0.9 and 1 in all cases, except when the test is not valid. In those cases, the maximum output probability value was found >0.7 in all cases: It was therefore decided to take 0.7 as the output probability threshold for both classes. That enables the app to train only on positive and negative RDT samples. An invalid RDT was defined as any result that did not show the control band. In our experience, this happened if an insufficient amount of buffer was applied (preventing the analyte from reaching the control band), or when the well was too small (creating clogs of blood preventing migration).

Using an app to help RDT users better confirm infection presents some strong advantages: Users learn how to perform valid tests through demonstration videos or blood sampling schematics included in the app. The app’s timer function facilitates timely reading (usually 7 to 15 min) decreasing the number of FPs. Results are independent of human error and subjectivity (up to 20% of one RDT presented faint, difficult-to-interpret bands; these represent the entirety of the FN samples in our experiments, where the ANN, despite having been trained on images with enhanced contrast, is unable to distinguish the bands from the background). The app displays results unambiguously (positive, negative, or invalid) without interpretation, translation errors, or jargon. App location data can direct users to local health services for medical advice. While the app and method could, in principle, be used with any generic RDT, it allows only for quality-controlled and locally authorized RDTs to be read. Since the devices’ sensitivities and specificities are known, the likelihood of a patient being positive is known. An added advantage is that the total number of the positive and negative tests is updated in real time, potentially providing an accurate evaluation of predictive values of each test. Finally, use of the app by health authorities using (fully anonymized) location data could produce live disease maps.

## Materials and Methods

Eleven antibody-detecting RDTs were chosen to evaluate the xRCovid app’s performance. Selected RDTs detected both total antibodies or IgG and IgM in blood, serum, or plasma and included: 1) Novel Coronavirus (2019-nCOV) Antibody IgG/IgM (Avioq Bio-tech); 2) BiosynexCOVID-19 BSS (Biosynex Swiss); 3) Biotime SARS-CoV-2 IgG/IgM Rapid Qualitative Test Kit (Xiamen Biotime Biotechnology); 4) NG-Test IgG-IgM COVID-19 cassette (NG-Biotech); 5) NG-Test IgG-IgM COVID-19 all-in-one (NG-Biotech); 6) 2019-nCoV IgG/IgM (Biolidics); 7) 2019-nCOV IgG/IgM Rapid Test Device (Realy Tech); 8) Nova COVID-19 IgG/IgM Rapid Test (Atlas Link); 9) Alco Digital COVID-19 IgG/IgM Rapid Test (Safecare Biotech); 10) SARS-CoV-2 IgG/IgM Rapid Test Solo Lab (Shuhai Encode Medical Engineering); and 11) COVID-19-CHECK-1 (Vedalab).

RDTs were chosen based on supply, availability, expected performance (per published literature), and information provided in commercial brochures. Tests were performed by trained laboratory technicians at room temperature according to the manufacturer’s instructions. All tests followed strict biosecurity measures and good microbiological practices and procedures (13).

To evaluate the xRCovid app performance, about 600 images of 600 SARS-CoV-2 RDTs were captured per hour for a total of 3,344 RDTs, in the following sequence: After a human reading by at least two analysts, the xRCovid app read the images in order. A stand was used to center all RDTs under the camera of an iPhone, elevating the phone to a distance of 98 mm from the reference plane on which the RDT was positioned (stand drawings as three-dimensional printable files are found in ref. 14). iPhone XR, XS, and SE (Apple) were used for all image captures during this field trial of the app. The images were kept on device and pushed to a cloud server for independent evaluation. As such, any image was read at least twice by human analysts and a third time if there was a mismatch between the first two readings. Source data used to train the neural network are found in ref. 14.

## Data Availability

Images/scripts have been deposited in GitHub (https://github.com/dmendels-collab/xRcovid) and Zenodo (https://zenodo.org/badge/latestdoi/312230700). All other study data are included in the article.
